# Phenotype prediction in plants is improved by integrating large-scale transcriptomic datasets

**DOI:** 10.1093/nargab/lqae184

**Published:** 2024-12-27

**Authors:** Zefeng Wu, Yali Sun, Xiaoqiang Zhao, Zigang Liu, Wenqi Zhou, Yining Niu

**Affiliations:** State Key Laboratory of Aridland Crop Science, Gansu Agricultural University, No. 1 Yingmen Village, Anning District, Lanzhou 730070, Gansu Province, China; State Key Laboratory of Aridland Crop Science, Gansu Agricultural University, No. 1 Yingmen Village, Anning District, Lanzhou 730070, Gansu Province, China; State Key Laboratory of Aridland Crop Science, Gansu Agricultural University, No. 1 Yingmen Village, Anning District, Lanzhou 730070, Gansu Province, China; State Key Laboratory of Aridland Crop Science, Gansu Agricultural University, No. 1 Yingmen Village, Anning District, Lanzhou 730070, Gansu Province, China; Crop Research Institute, Gansu Academy of Agricultural Sciences, No. 1, New Village, Anning District, Lanzhou 730070, Gansu Province, China; State Key Laboratory of Aridland Crop Science, Gansu Agricultural University, No. 1 Yingmen Village, Anning District, Lanzhou 730070, Gansu Province, China

## Abstract

Research on the dynamic expression of genes in plants is important for understanding different biological processes. We used the large amounts of transcriptomic data from various plant sample sources that are publicly available to investigate whether the expression levels of a subset of highly variable genes (HVGs) can be used to accurately identify the phenotypes of plants. Using maize (*Zea mays* L.) as an example, we built machine learning (ML) models to predict phenotypes using a gene expression dataset of 21 612 bulk RNA sequencing samples. We showed that the ML models achieved excellent prediction accuracy using only the HVGs to identify different phenotypes, including tissue types, developmental stages, cultivars and stress conditions. By ML models, several important functional genes were found to be associated with different phenotypes. We performed a similar analysis in rice (*Orzya sativa* L.) and found that the ML models could be generalized across species. However, the models trained from maize did not perform well in rice, probably because of the expression divergence of the conserved HVGs between the two species. Overall, our results provide an ML framework for phenotype prediction using gene expression profiles, which may contribute to precision management of crops in agricultural practices.

## Introduction

Although single-cell-based transcriptome sequencing technology is available, bulk RNA sequencing (RNA-Seq) technology still has an irreplaceable role because of its advantages of low cost, simple operation process and rapid data output. Advances in RNA-Seq technology have led to a wide range of large-scale gene expression studies that have provided an unprecedented amount of gene expression information (1). Thousands of plant RNA-Seq datasets have been deposited in public databases, such as the Sequence Read Archive, Gene Expression Omnibus and species-specific omics databases (2,3). Gene expression studies in plants have generated a wealth of transcriptomic data from different tissues, at different developmental stages, and/or under different stress conditions. These transcriptomic datasets are valuable resources for exploring the relationships between gene expression and phenotype, and statistical methods, such as cluster or differentially expressed gene analyses, have been widely used for this purpose (4,5). However, these methods are generally limited to small datasets and can hardly be used to make decisions when faced with a newly generated dataset. Therefore, integrated analysis of large-scale RNA-Seq datasets using appropriate statistical approaches is needed to model and understand the relationships between gene expression and phenotype.

Machine learning (ML)-based methods offer an alternative approach to explore how changes in gene expression lead to phenotypic changes, such as disease and health, and to enable data-driven biomarker discovery (6–9). Until now, most ML-based studies have been applied only to human cancer and other diseases, and their application in plants is still in its infancy. Several international consortia have focused mainly on human and other animals, and have provided resources that include the Encyclopedia of DNA Elements, Genotype-Tissue Expression and The Cancer Genome Atlas (10,11). Only a limited number of such unified resources are currently available for plants, including fruitENDODE (12), pENCODE (13) and riceENCODE (14). More recently, several plant gene expression databases have been developed by reanalyzing the public datasets using a uniform standard (2,3). These uniformly processed gene expression atlases provide the opportunity to explore the relationships between gene expression and phenotype in plants using ML approaches.

Maize (*Zea mays* L.) and rice (*Orzya sativa* L.), which diverged >50 million years ago, are economically important crops and primary sources of calories in human diets (15,16). Large-scale gene expression profiles have been generated for different tissues, developmental stages and various stress conditions in these two species (3). In this study, we applied an ML approach to a large-scale well-processed gene expression dataset of the two crops to model the relationship between the gene expression patterns and phenotype. Using only the highly variable genes (HVGs), the models accurately predicted tissue types, developmental stages, stress types and genotypes. We demonstrated that the predictive models were applicable across species using HVGs as features. By integrating the ortholog information, we also showed that the trained models from one of the species performed only moderately on data from the other species, likely because of differences in gene expression specificity between maize and rice. Overall, our results not only provide new insights into the complex relationships between gene expression and phenotype in plants, but also contribute to intelligent agricultural decision making, such as monitoring adverse stresses, determining developmental stages and identifying crop varieties.

## Materials and methods

### Gene expression dataset retrieval

Maize and rice gene expression profiles were downloaded from the PlantExp public database (3). Briefly, the maize gene expression data were downloaded from https://biotec.njau.edu.cn/plantExp/info.php?taxonId=4577, and the rice gene expression data were downloaded from https://biotec.njau.edu.cn/plantExp/info.php?taxonId=39947. The maize data consisted of 45 795 gene models (B73_RefGen_v4) and covered 21 612 RNA-Seq samples (Supplementary Data S1). The rice data consisted of 38 866 gene models (IRGSP-1.0) and covered 9965 RNA-Seq samples (Supplementary Data S2). Gene expression levels were quantified using TPM (transcripts per kilobase million) values.

### Identification of HVGs in maize and rice

Dropout-based feature selection was used to identify HVGs in the gene expression atlas by fitting the function between coefficient of variation squared (CV^2^) and mean expression (17,18). For this, the M3Drop R package was applied with the BrenneckeGetVariableGenes function, with maize and rice gene expression profile data as the inputs (18). We obtained 2880 HVGs for maize using the default parameters. More than one-third of the protein coding genes in rice were identified as HVGs using the default parameters. To compare the maize and rice data, we set a stricter fitMeanQuantile parameter of 0.4 for rice, and obtained 3997 rice HVGs.

### ML model building

ML models were used to analyze the RNA-Seq data and fit the relationship between gene expression and sample source. We used five ML models to build classifiers, namely Random Forest, Support Vector Machine, Naive Bayes, XGBoost and Deep Neural Network. The Random Forest model was built using the randomforest function implemented in the randomForest R package with the default parameters (https://CRAN.R-project.org/package=randomForest). The Support Vector Machine model was run using the svm function with parameters type=‘C’ and kernel=‘linear’ implemented in the e1071 R package (https://CRAN.R-project.org/package=e1071). The other kernel functions were also used, but the ‘linear’ one was found to have the best performance. The Naive Bayes model was run using the naiveBayes function with the default parameters implemented in the e1071 R package. The XGBoost model was run using the xgboost R package with parameters max_depth = 6, eta = 0.5 and objective=‘multi:softmax’ based on the results of a serious of hyperparameters selection. The Deep Neural Network model was built with two denser layers using the Keras and Tensorflow R packages (https://CRAN.R-project.org/package=keras). The gene expression profile data were randomly divided into training and test datasets with a ratio of 7:3, and this random sampling was performed five times to exclude model overfitting. After training the models on the training dataset, the model performance was measured on test data by calculating a confusion matrix and area under the receiver operating characteristic curve (AUC) values using the caret (https://CRAN.R-project.org/package=caret) and pROC (https://CRAN.R-project.org/package=pROC) packages, respectively.

### Identification of orthologous genes

Orthologous genes between maize and rice were identified using the reciprocal BLAST implemented in the metablastr R package (https://github.com/drostlab/metablastr). The protein sequences of maize (RefGen_v4) and rice (IRGSP-1.0) were retrieved from the Ensembl Plants database (19). When a gene encoded multiple transcripts, the longest protein sequence was retained for the further analysis. Reciprocal BLAST was performed with e-value cutoff of 1e−5.

### Tissue-specific expression of conserved HVGs in maize and rice

For each conserved HVG (cHVG) from maize and rice, the tissue-specific expression patterns of the gene were investigated using two dependent approaches. As there were multiple samples for each tissue type in the maize or rice data, we used the median gene expression value for each cHVG across different samples as the gene expression level in each tissue. In one method, the tissue expression specificity for each cHVG was recorded by selecting the tissue in which the gene was most highly expressed. Then, the tissue expression specificity of each cHVG was compared between maize and rice. In the other method, the Pearson correlation coefficient was calculated for each pair of cHVGs from maize and rice, based on the summarized gene expression level in different tissues of the two species.

### Gene functional enrichment analysis

Maize gene ontology information was retrieved from the agriGO database (20). Functional enrichment analysis of the maize HVGs was performed using the clusterProfiler R package with qvalueCutoff = 0.05 (21). Function enrichment analysis of rice HVGs was performed using the gprofiler2 R package with the default parameters (22).

## Results

### Selection of HVGs from a gene expression atlas

**Figure 1.**
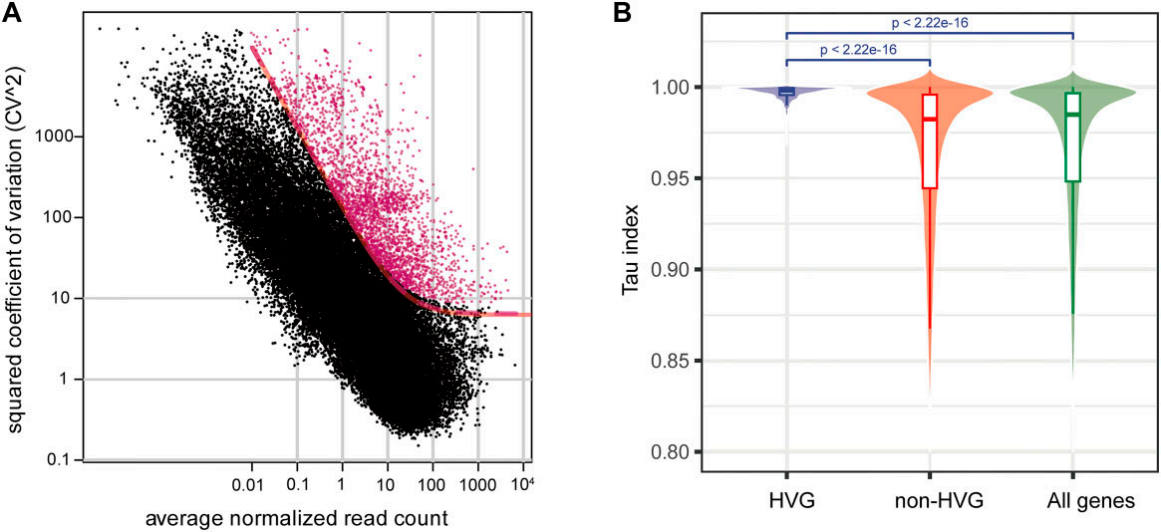
Characterization of maize HVGs based on gene expression profiles. (**A**) Identification of maize HVGs using the gene expression profiles from 21 612 RNA-Seq samples. Magenta, genes with coefficients of biological variation that are significantly higher than those of the controls. (**B**) Gene expression specificity by tau score between maize HVGs, non-HVGs and all protein-coding genes. Significant difference was calculated using the Wilcoxon rank sum test.

Feature selection is an important step in building an ML model (23), especially for high-dimensional biological data such as gene expression profiles, which consist of thousands of genes from different tissues, accessions or conditions. Unlike stably expressed genes, HVGs exhibit pronounced expression changes across multiple samples and tend to be more meaningful in explaining differences between samples in a gene expression atlas (24). Therefore, HVGs are likely to be important predictors or features for training ML models. Dropout-based feature selection is considered a robust approach to identify HVGs in single-cell RNA-Seq by fitting the function between the CV^2^ and mean expression, which ensures that a fraction of HVGs can be obtained from any set of genes with different expression levels (17). We used this method and identified 2880 HVGs in the maize datasets (Figure 1A, and Supplementary Data S3; see ‘Materials and methods’ section). Gene function enrichment analysis showed that these HVGs were involved mainly in killing of cells of other organisms, lipid transport and sexual reproduction (Supplementary Figure S1). To determine whether these HVGs were expressed in a tissue-specific manner, we calculated the tau index of gene tissue specificity. We found that the HVGs had significantly higher tau values than the controls had (Figure 1B), suggesting that the maize HVGs may potentially be marker genes in specific tissues, at specific developmental stages, or under specific stress conditions (25).

### Tissue type prediction based on HVGs

**Figure 2.**
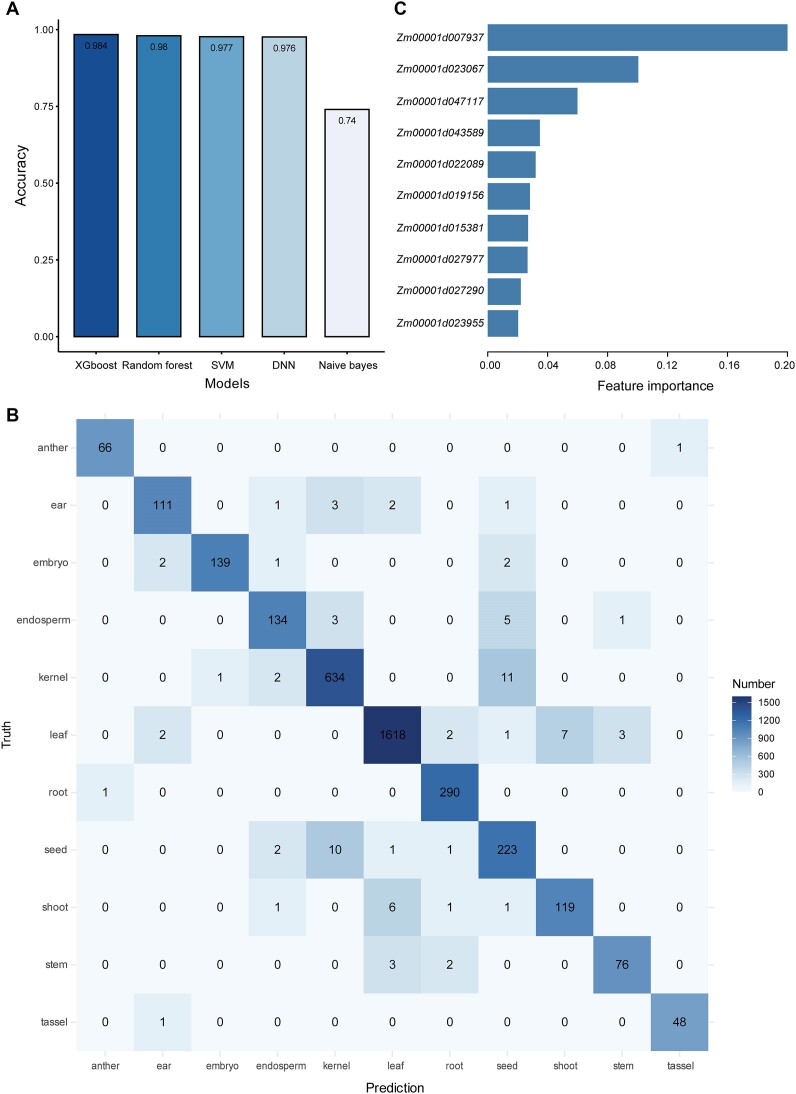
Prediction of tissue type based on HVG expression levels in maize. (**A**) Prediction accuracy of five ML models. SVM: support vector machine; DNN: deep neural network. (**B**) Confusion matrix of the predicted tissue types obtained using the XGBoost model versus true tissue types. (**C**) Bar graph showing the gain-based feature importance of the XGBoost model. The top 10 genes are shown.

To investigate whether HVGs can be used to accurately predict sample sources, we compiled a maize gene expression dataset consisting of 11 795 samples that covered 11 typical maize tissues: leaf, root, kernel, seed, endosperm, shoot, stem, ear, anther, embryo and tassel (Supplementary Figure S2). These tissues were chosen because enough maize sequencing samples for ML modelling were available. We built five ML models—Random Forest, Support Vector Machine, Naive Bayes, XGBoost and Deep Neural Network—to fit the tissue types and HVG expression levels (Supplementary Figure S3). Four of the models had predication accuracies >0.97; the exception was the Naive Bayes model, which had a prediction accuracy of 0.74 (Figure 2A). Among the four models with high predication accuracies, the XGBoost model had slightly higher prediction accuracy than the other three models, demonstrating its advantages in gene expression analysis. Similar prediction accuracies were obtained using AUC values to evaluate the performances of the five models (Supplementary Figure S4), further supporting the proposal that HVG expression levels could accurately distinguished tissue types in maize. We analyzed the confusion matrix derived from the XGBoost model and found that most of the misclassifications were from pairs of tissues with an inclusive relationship or similar physiological characteristics, such as kernel and seed, and leaf and shoot (Figure 2B). Our analysis of the feature importance of the HVGs showed that five genes—*Zm00001d007937* (*alanine amino transferase 8*), *Zm00001d023067*, *Zm00001d047117* (*physical impedance induced protein1*), *Zm00001d043589 (MADS36)* and *Zm00001d022089 (ZmEA1)*—had higher prediction importance than the other HVGs based on the average gain of a feature over all trees for the XGBoost prediction results (Figure 2C). The feature importance of these HVGs is consistent with the high gene expression variation and specificity of gene expression in different tissues (Supplementary Figure S5); for example, *Zm00001d007937*, *Zm00001d022089*, *Zm00001d047117* and *Zm00001d023067* were highly expressed in leaf, embryo, root and kernel, respectively.

To rule out the possibility that the structure of the HVG expression profile data effected the model building, two independent simulation analyses were performed by randomly shuffling HVG expression or by randomly sampling an equal number of genes from the original gene expression profiles. By randomly shuffling HVG expression, the performances of all five ML models were weak, with prediction accuracy of only up to 0.46 (Supplementary Figure S6A). Unexpectedly, all five models produced good prediction accuracies by randomly sampling an equal number of genes from the original gene expression profiles (Supplementary Figure S6B). This result suggests that there was gene expression redundancy between HVGs and non-HVGs in determining phenotypes in maize. Therefore, we concluded that using HVGs as predictive features is a sufficient but not a necessary condition for tissue type prediction in maize. However, considering that HVGs generally provide more information than stably expressed genes in understanding gene dynamic expression, we used mainly the HVGs in the subsequent analyses.

Considering that the HVGs in the above analysis were selected from the whole dataset, which included both the training and test datasets, the high prediction accuracy could be the result of data leakage. To exclude this possibility, the HVGs were selected from the training dataset only, and the different ML models were trained to predict tissue types in the test dataset. As a result, all the models had good predictive performance with accuracies of 0.79–0.98 (Supplementary Figure S7), which were close to or even better than the models using HVGs from the whole dataset. Accordingly, the excellent of prediction results were unlikely to be the cause of a data leakage. Since the identification of HVGs depended on the number of gene expression samples, we used the HVGs selected from the whole dataset in the following analysis.

### Development stage modelling using HVGs

**Figure 3.**
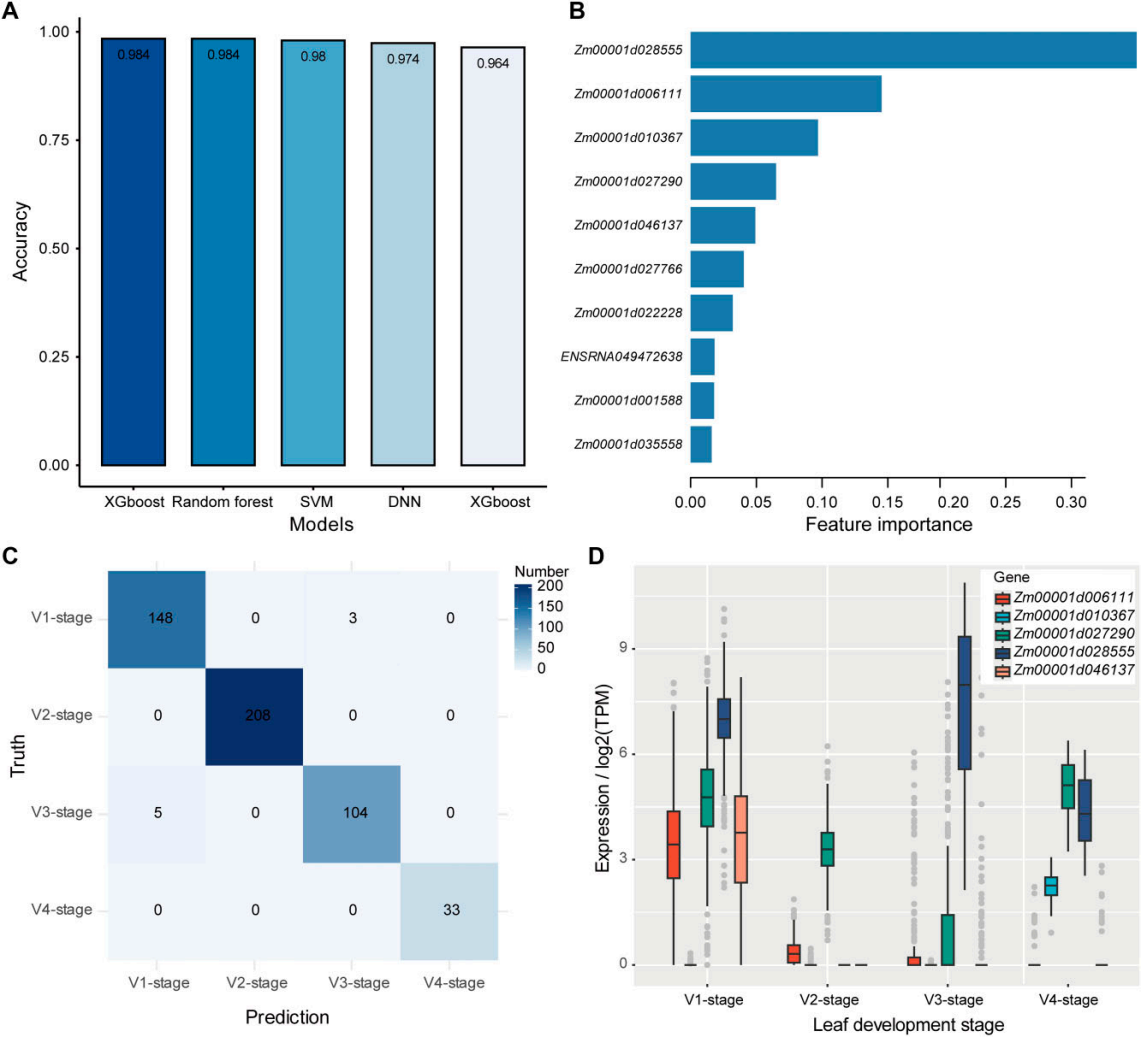
Prediction of leaf development stages based on HVG expression in maize. (**A**) Prediction accuracy of five ML models. SVM: support vector machine; DNN: deep neural network. (**B**) Bar graph showing the gain-based feature importance obtained using the XGBoost model. The top 10 genes are shown. (**C**) Confusion matrix showing the predicted leaf developmental stage obtained using the XGBoost model and actual leaf developmental stage. (**D**) Expression levels of the top five features (HVGs) that were highly dynamically expressed at four leaf development stages obtained using the XGBoost model.

To investigate whether the developmental stages of maize can be modeled based on the HVG expression information, we compiled a dataset of 501 leaf samples at four developmental stages: V1, V2, V3 and V4, where ‘V*n*’ represented different vegetative stages. Using the HVGs as features, we trained the five ML models using the training data. The results showed that all five models identified most of the tested samples with prediction accuracies of 0.96–0.98 and AUC values of 0.98–0.99 (Figure 3A, and Supplementary Figure S8). Feature importance analysis identified *Zm00001d028555 (HSP20)*, *Zm00001d006111 (CYBDOM)*, *Zm00001d010367* (uncharacterized protein), *Zm00001d027290* (extensin-like protein) and *Zm00001d046137* (uncharacterized protein) as the top five HVGs that determined the leaf development stage in maize (Figure 3B). The high predictive accuracy was also confirmed by the confusion matrix (Figure 3C). HVG expression analysis showed that these genes had highly dynamic expression at different leaf development stages (Figure 3D). For example, *Zm00001d028555* was highly expressed at V1 and V3, *Zm00001d006111* and *Zm00001d046137* were highly or specifically expressed at V1, *Zm00001d010367* was specifically expressed at V4 and *Zm00001d027290* was highly expressed at V1 and V4 (Figure 3D).

### Identification of different maize inbreds using HVGs

**Figure 4.**
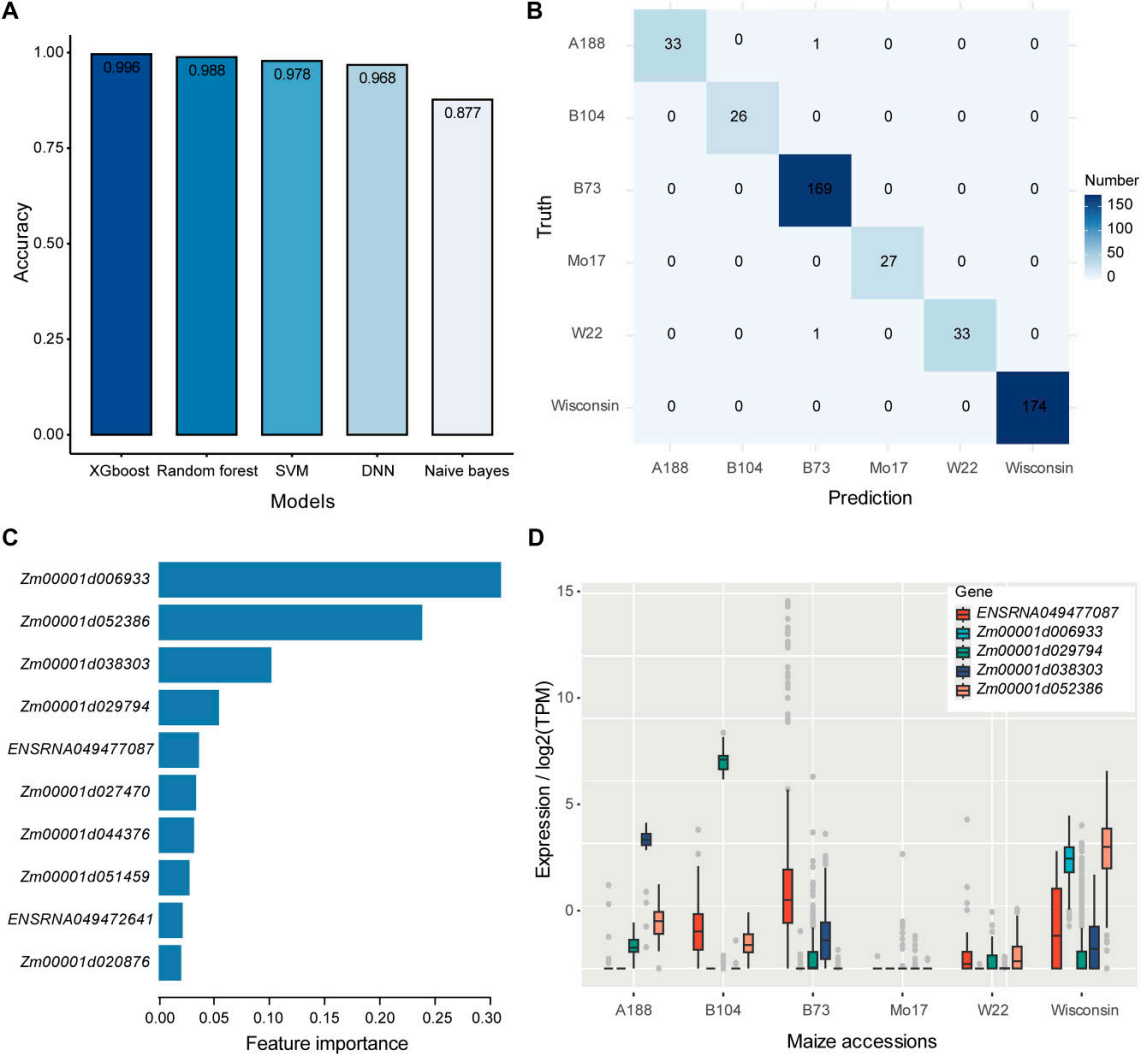
Prediction of maize inbreds based on HVG expression in maize. (**A**) Prediction accuracy of five ML models. SVM: support vector machine; DNN: deep neural network. (**B**) Confusion matrix showing the predicted maize inbreds obtained using the XGBoost model and true maize inbreds. (**C**) Bar plot showing the gain-based feature (HVG) importance obtained using the XGBoost model. The top 10 genes are shown. (**D**) Expression level of the top five features (HVGs) in the different maize inbreds obtained using the XGBoost model.

Genetic variation has been shown to affect tissue-specific gene expression (26,27). To investigate differences in transcriptomic changes in different maize inbreeds or accessions, we trained the five ML models to fit HVG expression levels and maize accessions. First, we compiled a dataset of 1546 samples that covered six maize accessions, namely A188, B104, B73, Mo17, W22 and Wisconsin. Then, we used the HVGs to train the five ML models and evaluated their prediction accuracies. The results showed that all the models had good predictive power with accuracies of 0.87–0.99. Among them, the XGBoost model again had the best performance (Figure 4A) with only two misclassified samples in the tested dataset; one A188 and one W22 sample were both predicted to be B73 samples (Figure 4B). Feature importance analysis showed that *Zm00001d006933* (*PER2_19*), *Zm00001d052386* (encoding a putative apyrase family protein), *Zm00001d038303* (uncharacterized protein), *Zm00001d029794 (chitinase)* and *ENSRNA049477087* (plant_SRP) were the top five genes that discriminated the different maize inbreds (Figure 4C). The HVG expression profiles showed that these genes had high accession-specific expression patterns. For example, *Zm00001d006933* and *Zm00001d052386* were specifically or highly expressed in Wisconsin, whereas *Zm00001d038303* was highly expressed in A188 and not expressed or lowly expressed in the other maize accessions (Figure 4D). *Zm00001d029794*, which encodes a chitinase, was highly expressed in B104, and *ENSRNA049477087* was highly expressed in B73 (Figure 4D). These accession-specific expressed genes may be important markers for identifying maize accessions.

### HVGs are indicators of stress types

**Figure 5.**
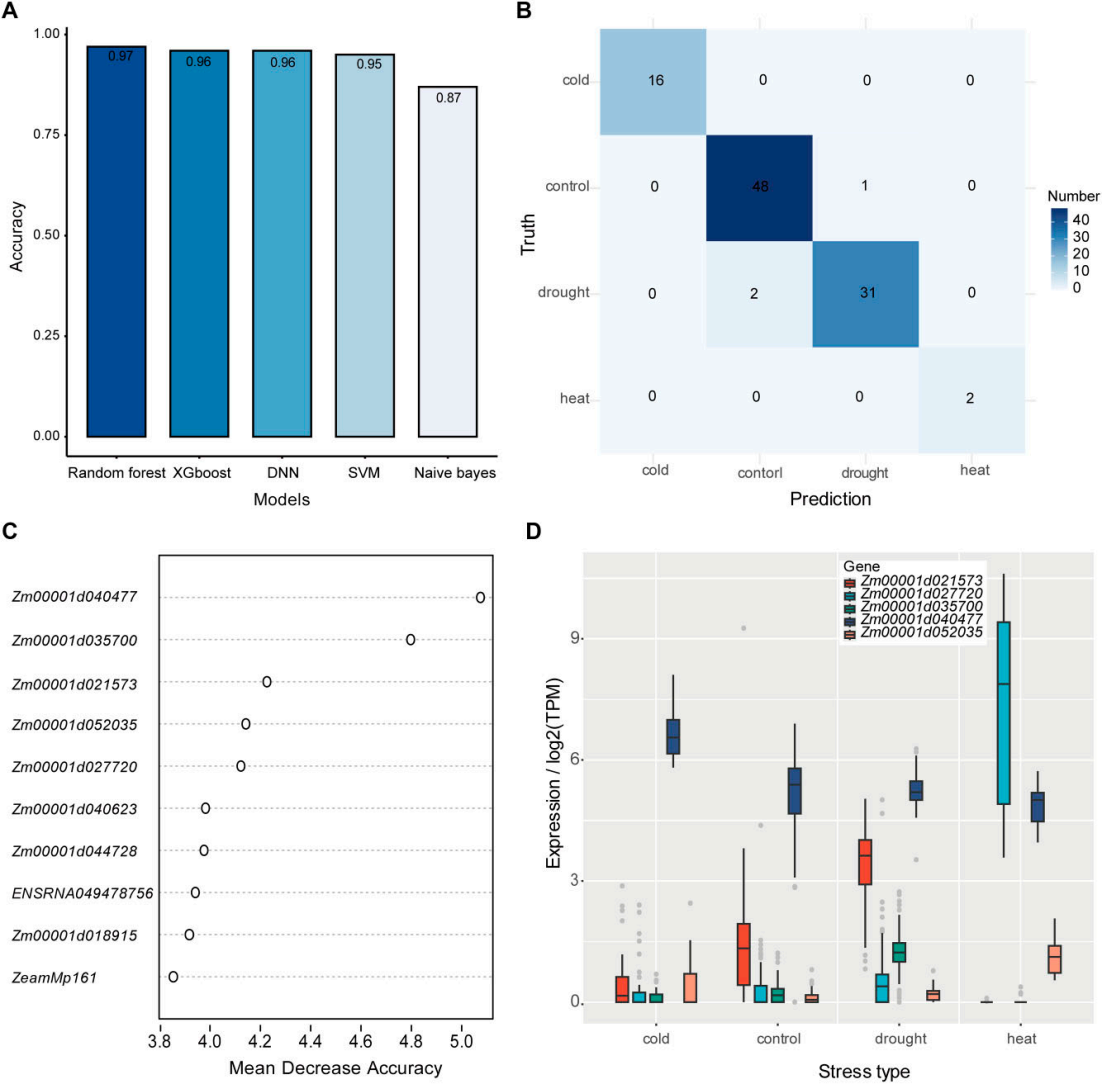
Prediction of stress type based on HVG expression in maize. (**A**) Prediction accuracy of five ML models. SVM: support vector machine; DNN: deep neural network. (**B**) Confusion matrix showing the predicted stress types obtained using the Random Forest model and the true stress types. (**C**) Dot plot showing the mean decrease-based feature (HVGs) importance obtained using the Random Forest model. The top 10 genes are shown. (**D**) Expression levels of the top five features (HVGs) in maize under different stresses obtained using the Random Forest model.

Accurate identification of potential adverse stresses experienced by plants is essential for precise crop management (28,29). To explore potential molecular markers that could be indicators of the types of adverse stresses experienced by maize, we compiled a maize stress dataset. Although many publicly available maize RNA-Seq datasets have been generated under stress conditions, they were not focused on a specific tissue under a specific stress condition. Indeed, we obtained only one unified dataset of 333 RNA-Seq leaf samples covering three types of abiotic stresses, namely drought, heat and cold, and their controls. We used this dataset to train the five ML models and evaluated their performances. The prediction accuracies of the five ML models were 0.87–0.97 (Figure 5A), suggesting that the HVGs fully identified the stress types experienced by maize. Among the five models, the Random Forest model performed slightly better than the other four models. Confusion matrix analysis of the Random Forest results showed that only three samples were misclassified between the drought and control samples (Figure 5B). Feature importance analysis showed that, in the Random Forest model, *Zm00001d040477*, *Zm00001d035700*, *Zm00001d021573*, *Zm00001d052035* and *Zm00001d027720* were the top five features for predicting stress type in maize (Figure 5C), indicating their potential value in determining stress types. These five HVGs consistently exhibited a stress-type-dependent expression pattern (Figure 5D). For example, *Zm00001d040477*, which encodes an F-box domain protein, was highly expressed under cold stress, followed by control, drought and heat stress. *Zm00001d035700*, which encodes legumin1 (ZM-LEGF), was highly expressed under drought stress. The detailed molecular functions of these genes in the response to drought stress require further research. *Zm00001d021573*, encoding a SPB-box transcription factor, was highly expressed under drought stress, followed by the control, cold stress, and lowly expressed under heat stress. *Zm00001d052035*, encoding RNA polymerase II C-terminal domain phosphatase-like 4, was highly expressed under heat stress and relatively lowly expressed under the other stresses and in the control. *Zm00001d027720*, encoding a heavy metal-associated isoprenylated plant protein 27, was significantly induced under heat stress, suggesting that it may be an important marker gene in the response to high temperature. The feature importance analysis of the XGBoost model identified two genes in the top five features, *Zm00001d035700* and *Zm00001d040477*, which overlapped with genes identified by the Random Forest model (Supplementary Figure S9A). This result further suggested the relatedness of these two genes with abiotic stress in maize. We obtained other important features using the XGBoost model in addition to those that overlapped with those using the Random Forest model. For example, *Zm00001d028408*, which encodes the widely studied heat shock protein HSP26 (30,31), was extremely highly induced under heat stress (Supplementary Figure S9B). *ENSRNA049478756* and *ENSRNA049461556* encode eukaryotic small subunit ribosomal RNA and small nucleolar RNA snoR97, respectively, and both were induced under all surveyed stress conditions, suggesting these non-coding RNAs may be involved in the stress response (Supplementary Figure S9B).

### Generalization of models across species

**Figure 6.**
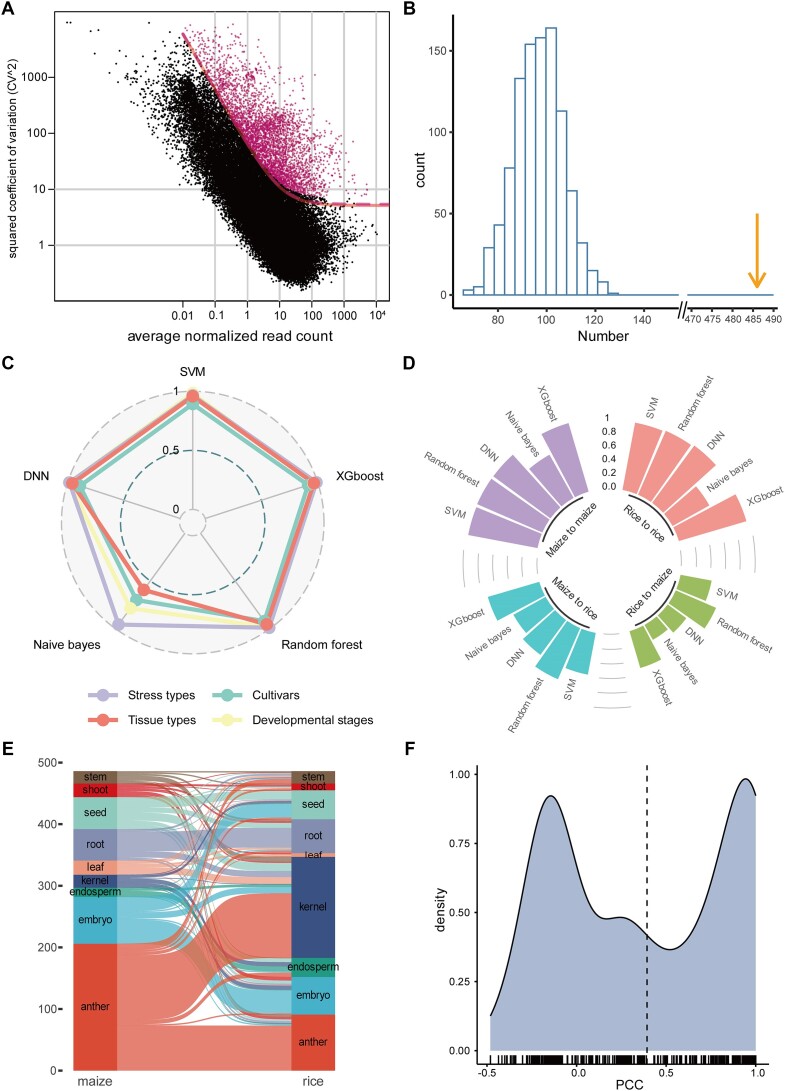
Comparison of prediction models between maize and rice. (**A**) Identification of HVGs in rice using gene expression data from 9965 RNA-Seq samples. (**B**) Histogram showing the number of cHVGs between maize and rice from 1000 simulations. The orange arrow indicates the actual number of the HVGs shared between the two species. (**C**) Radar plot showing the prediction accuracy of the five models for tissue types, stress types, developmental stages and varieties. SVM: support vector machine; DNN: deep neural network. (**D**) Circle bar plot showing the performance of the models for predicting tissue types. The models were trained on both maize and rice data, and performance was evaluated on maize and rice using the model trained on either the same or the other species. (**E**) Sankey plot showing the gene expression preference of the cHVGs in maize and rice. (**F**) Distribution of the gene expression correlation between all pairs of cHVGs from the two species. PCC: Pearson correlation coefficient.

To test the degree of generalization of the prediction models across species, we performed similar analyses in rice using the same methods that we used on the maize datasets with the default settings. More than one-third of the rice genes were identified as HVGs (Supplementary Data S4). To compare the rice data with the maize data, we adjusted the threshold and obtained 3887 rice HVGs (Figure 6A, and Supplementary Data S5). By homologous comparison analysis, we found a significantly high proportion of HVGs that overlapped between maize and rice (Figure 6B), suggesting that HVGs were relatively conserved in plant evolution. The gene tissue specificity of rice HVGs was measured using the tau index, and the results were similar to those obtained for maize HVGs (Supplementary Figure S10). Interestingly, the gene function enrichment analysis of rice HVGs showed that some of the enriched gene ontology terms under the biological process category, including hydrogen peroxide catabolic process, polysaccharide metabolic process and sexual reproduction, overlapped with those of maize. Similar results were obtained for the enriched terms under the molecular function and cellular component categories (Supplementary Figure S11), suggesting the functions of the HVGs were conserved across species.

Next, we used the rice HVGs to model the relationship between gene expression and tissue types using the five ML models. All the models performed well (Figure 6C), although their performances were slightly lower than those for the maize HVGs. The different developmental stages, cultivars and stress types of rice were also well predicted (Figure 6C), further demonstrating that phenotypes were predicted successfully using HVG expression in different plant species.

Then, we investigated whether the prediction models trained in one species could be used for prediction in another species. To this end, we used the 486 cHVGs shared by maize and rice. Because not enough RNA-Seq datasets that matched accessions, developmental stages or stress conditions between the two species were available, we focused only on predicting RNA-Seq-derived tissue types. We trained the five models for tissue type prediction using the maize expression profiles of the 486 cHVGs. The performance analysis showed that the prediction accuracy of the models was slightly reduced but still very close to that using the 2880 maize HVGs (Figure 6D), indicting redundancy among the HVGs. We applied the trained models from maize to predict the tissue types in the matched rice dataset. Compared with the model performances in maize, the prediction accuracies of the models in rice were relatively low (0.50–0.76) (Figure 6D). We also trained the models in rice using the 486 cHVGs, and obtained high prediction accuracies that were comparable to those using the 3887 rice HVGs (Figure 6D). Using the models trained in rice, we predicted the tissue types in the matched maize dataset. We found that the prediction accuracies of the models were only 0.23–0.60 (Figure 6D). We reasoned that the unequal performances of the intraspecies and interspecies models may be because the cHVGs have different tissue-specific expression patterns in the two species.

To confirm this hypothesis, we compared the gene expression patterns of the 486 cHVGs in maize and rice. Each cHVG was deemed to show tissue-specific expression if the cHVG was most highly expressed in a particular tissue type. Then, the tissue specificity of the tissue-specific cHVGs was compared between maize and rice (Figure 6E). Interestingly, a moderate proportion of cHVGs had consistent tissue-specific expression patterns in the two species, especially those that were expressed mainly in root, embryo, endosperm and anther. However, some cHVGs showed distinct expression specificity between the two species. For example, many cHVG that were highly expressed in maize leaf or anther were expressed mainly in rice kernel, suggesting frequent shifts or turnovers of gene expression specificity after the two species diverged. Notably, many of the turnovers occurred between pairs of closely related tissues, such as embryo and seed, embryo and kernel and kernel and endosperm. (Figure 6E). Whether these shifts are evolutionarily significant or simply caused by the confounding effect of the RNA-Seq tissue dissection requires further research. The relatively low conservation of the cHVG tissue-specific expression was also demonstrated by calculating the gene expression correlation between maize and rice across different tissues (Figure 6F). In general, the expression correlation of cHVGs had a bimodal distribution with two peaks corresponding approximately to 0.95 and −0.15, suggesting that both conservation and specificity of cHVG expression patterns occurred in maize and rice. The specificity of expression patterns may lead to relatively low accuracy when cHVGs are used to predict tissue types in one species using the models trained from another species.

## Discussion

Understanding the relationship between genotype and phenotype is a fundamental task in systems biology (32,33). RNA-Seq technology has provided rich gene expression atlases in plants, which has enabled the dynamic expression of genes to be studied in particular tissues, developmental stages or as a treatment (34). In this study, we applied ML methods to predict phenotypes using the large number of gene expression profiles from plant RNA-Seq data. We showed that using only the expression profiles of a subset of genes (i.e. HVGs), the ML models accurately predicted the phenotypes in maize and rice.

Among high-dimension gene expression profiles there is a lot of redundancy because of, for example, gene co-expression and protein–protein interactions (35,36). Therefore, extracting important genes or features can help to elucidate the regulation of gene expression in a particular condition. In general, HVGs strongly contribute to tissue-to-tissue expression variation in a homogeneous tissue, and this feature has been widely used to infer cell types in single-cell RNA-Seq (17). We identified 2880 and 3997 HVGs using dropout-based feature selection in large gene expression datasets from maize and rice, respectively. These HVGs exhibited high expression variation among different samples and had strong tissue-specific gene expression (Figure 1A), suggesting that they were potential predictors for identifying sample properties in the two species. Indeed, using these HVGs as the main predictive features, we trained five different ML models to predict the relationships between HVG expression and phenotype, resulting in a good fit with all the five models. When these models were trained on a small fraction of the samples, the tissue types, developmental stages, cultivars or adverse stresses experienced in test gene expression dataset were accurately predicted.

We also showed that the XGBoost model outperformed the other four ML models in most cases, suggesting that the XGBoost model is most suitable for processing large amounts of gene expression data. Several other studies have also shown that the XGBoost model is an efficient approach to modelling gene expression. For example, using gene expression levels of a small fraction of landmark genes, the XGBoost model successfully predicted the expression levels of the remaining genes (37). In another study, using evolutionarily conserved nitrogen-responsive genes, the XGBoost model accurately predicted the phenotypic diversity of nitrogen use efficiency in Arabidopsis and maize (38). Although deep learning-based models have been shown to perform well in solving complex biological problems, such as predicting gene expression levels, alternative splicing of genes and other biological signals (39–41), we did not find any advantages in the present research, which is consistent with the finding of a previous study (42). This may be because deep learning models for classification problems generally require a large number of samples for model training. In our study, there is data imbalance between the different phenotypes to be predicted. For example, when predicting tissue types, the number of samples for each tissue varied widely. Similarly, when predicting developmental stages and stress types, the sample numbers varied widely.

As expected, the relationship between HVG expression and phenotype was generalized across maize and rice. However, when the HVGs obtained using models trained on one of the species were used to evaluate the performance of the models on the other species that was not used for training, the accuracy of the models was relatively low. This result may be because of differences in gene expression specificity between the two species. Indeed, the gene expression specificity analysis showed that some cHVGs had different tissue expression preferences in maize and rice, implying gene expression evolved as species diverged. Some studies that focused on gene expression divergence in animals or plants showed that genes were subjected to the effects of purifying selective constraint or influenced by positive selection during species evolution (43,44). Divergence in gene expression between two species may also contribute to phenotypic novelty, such as the emergence of novel cell types (45,46).

Our study has some limitations mainly because of the limited availability of experimental measurements of plant transcriptomes under specific conditions. For example, only three types of stress conditions were available for leaf tissues in the current maize database, and there was not enough data for other types of stress in other tissues, which limited the use of the ML models. The cost of sequencing continues to decrease, and therefore increasing amounts of plant-related transcriptome data under specific conditions are likely to become available; for example, time-course data may be generated by the many international plant omics projects. Building ML models by combining the gene expression data and other omics data will undoubtedly promote crop management and agricultural decisions with higher precision.

## Supplementary Material

lqae184_Supplemental_Files

## Data Availability

Gene expression information of maize and rice used in this study can be accessed in a permanent FigShare repository: https://figshare.com/s/881d86b2f564a8ab905a.

## Code availability

Source code is available at https://github.com/Zefeng2018/plant-phenotype-prediction-by-gene-expression and https://doi.org/10.5281/zenodo.14358186.
