# Supplementary material for: Phenotype prediction in plants is improved by integrating large-scale transcriptomic datasets
Source: NAR Genom Bioinform. 2024 Dec 27;6(4):lqae184. doi: 10.1093/nargab/lqae184 (PMC11672113; doi:10.1093/nargab/lqae184)
Supplement: lqae184_Supplemental_Files [file lqae184_supplemental_files.zip › Supplementary figures.docx]

## Supplementary figures

## Phenotype prediction in plants is improved by integrating large-scale transcriptomic datasets

Zefeng Wu^1^, Yali Sun^1^, Xiaoqiang Zhao^1^, Zigang Liu^1^, Wenqi Zhou^2^, Yining Niu^1*^

^1^State Key Laboratory of Aridland Crop Science, Gansu Agricultural University, Lanzhou, 730070, Gansu, China

^2^Crop Research Institute, Gansu Academy of Agricultural Sciences, Lanzhou, 730070, Gansu, China

^*^Corresponding author: niuyn@gsau.edu.cn

Running title：Phenotype prediction by transcriptomic datasets


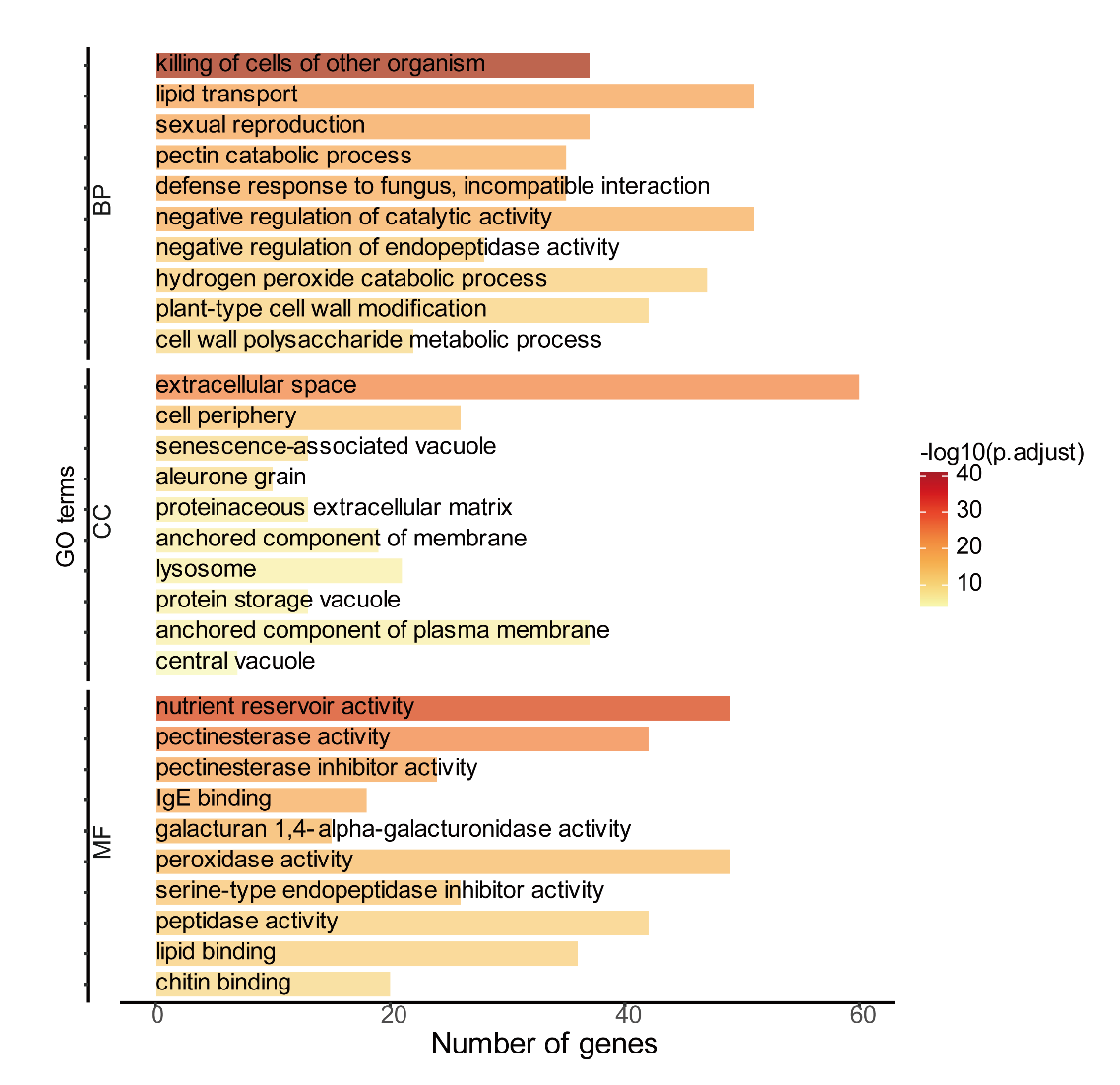


**Fig. S1 Gene function enrichment analysis of maize HVGs.** HVG, highly variable gene.


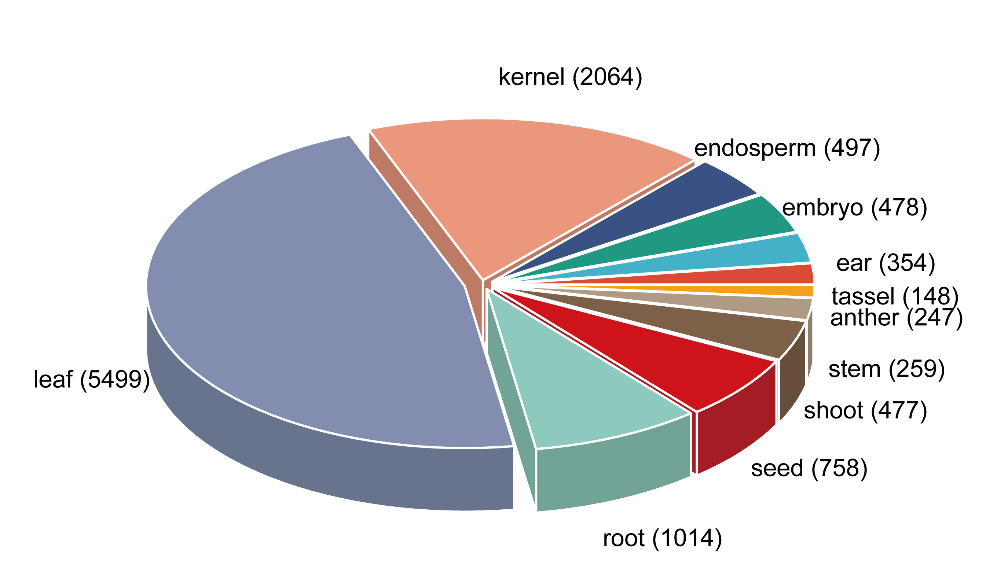


**Fig. S2 Pie chart showing the number of maize RNA-Seq samples from different tissues.**


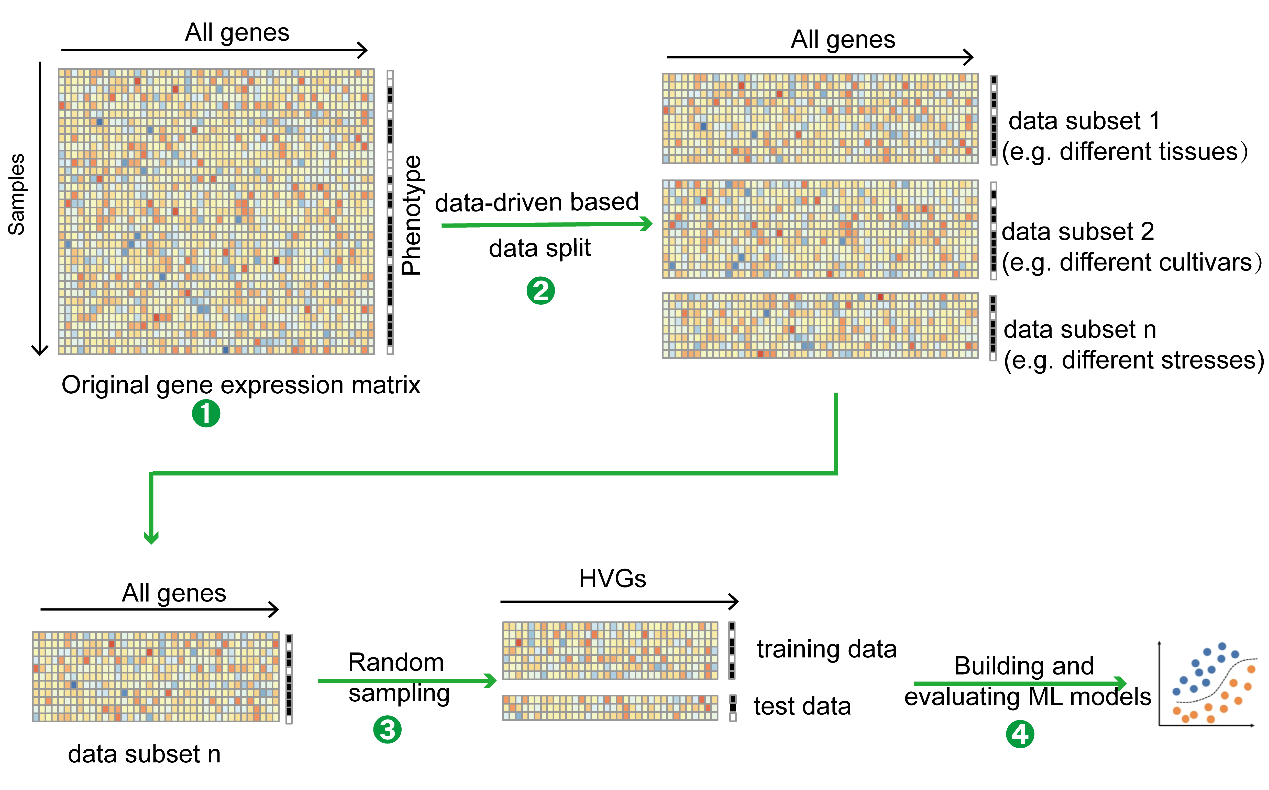


**Fig. S3 Workflow of phenotype prediction based on gene expression of HVGs.** The entire workflow consists of four main steps. First, the plant gene expression matrix is obtained from the public database. Each row in the matrix represents a sample, each column represents a gene, and the corresponding value in the matrix is the gene expression level. At the same time, the phenotype data of each sample was also obtained. Then, based on the sample phenotype, the whole matrix was divided into different subsets. For each subset, random sampling was performed to form training and test data. The HVGs identified from either the original gene expression matrix or the subset were used as predictive features to build and evaluate ML models for plant phenotype prediction.


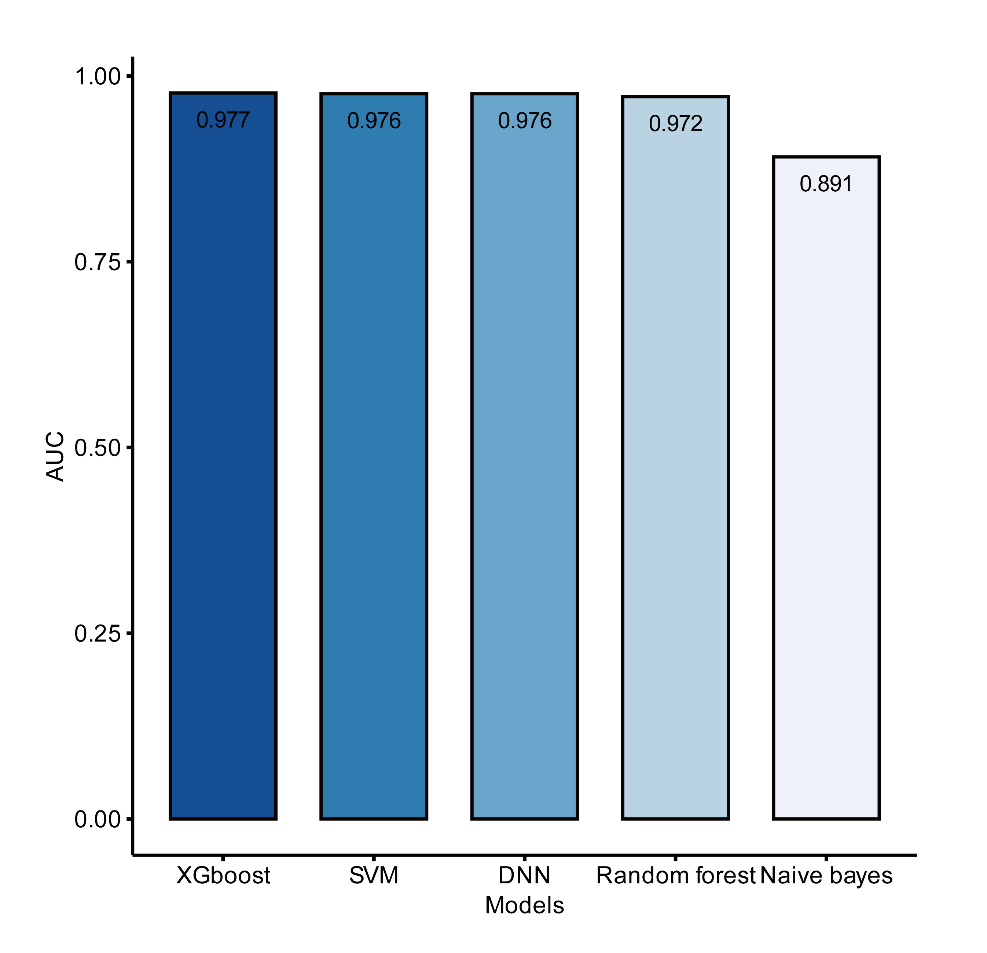


**Fig. S4 Evaluation of the model performance for tissue type prediction based on AUC values.**


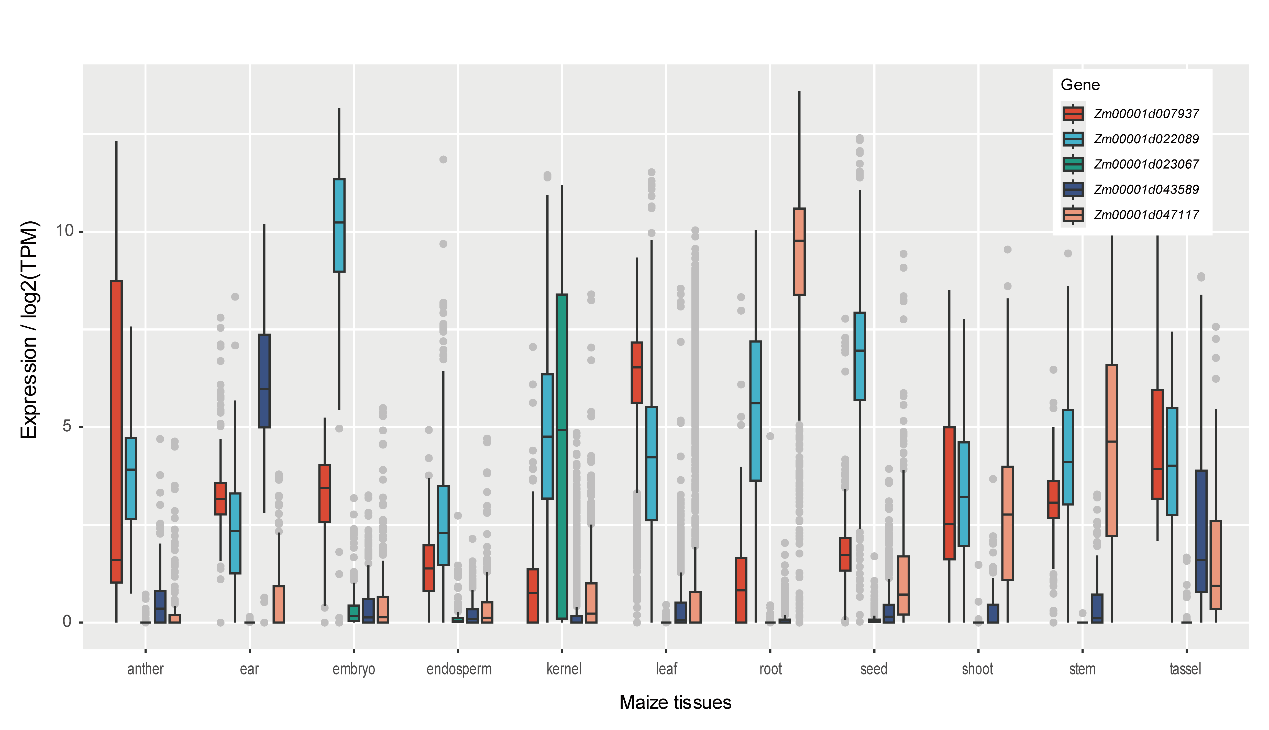
 **Fig. S5 Gene expression specificity of the top five features in predicting maize tissue types.**


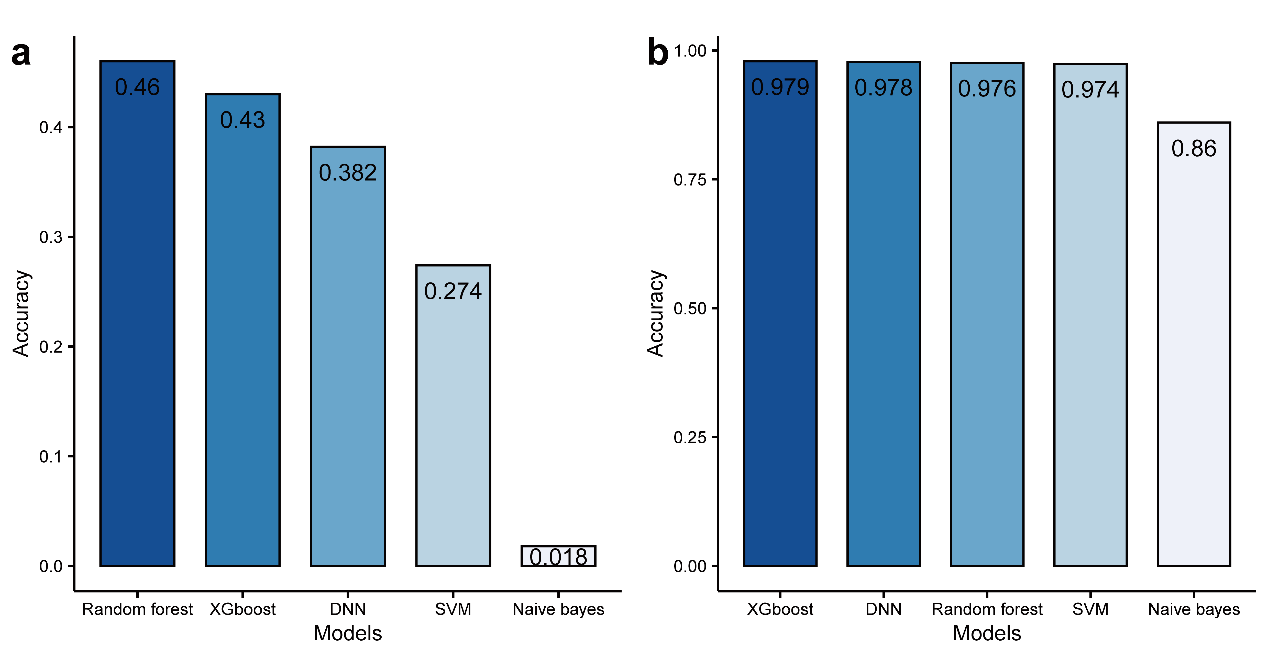


**Fig. S6 Model performance using a shuffled gene expression of maize HVGs.** **(a)** Model performance using a shuffled gene expression of HVGs in maize. **(b)** Model performance using a set of randomly sampled genes as HVGs. HVG, highly variable gene.


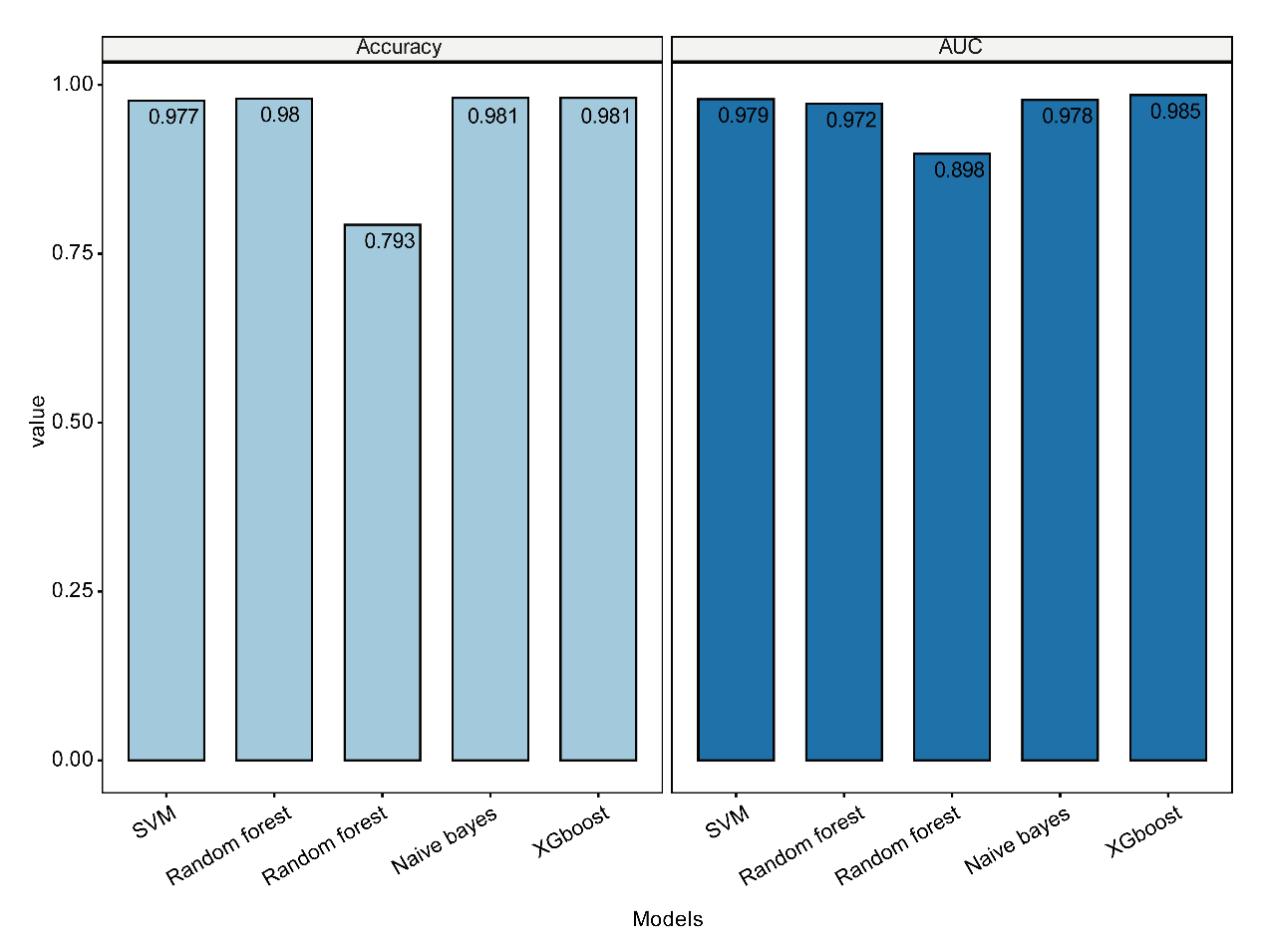
**Fig. S7 Model performance using maize HVGs selected from the training dataset.** Left panel shows the model performance measured by accuracy, and the right panel shows the model performance measured by AUC.


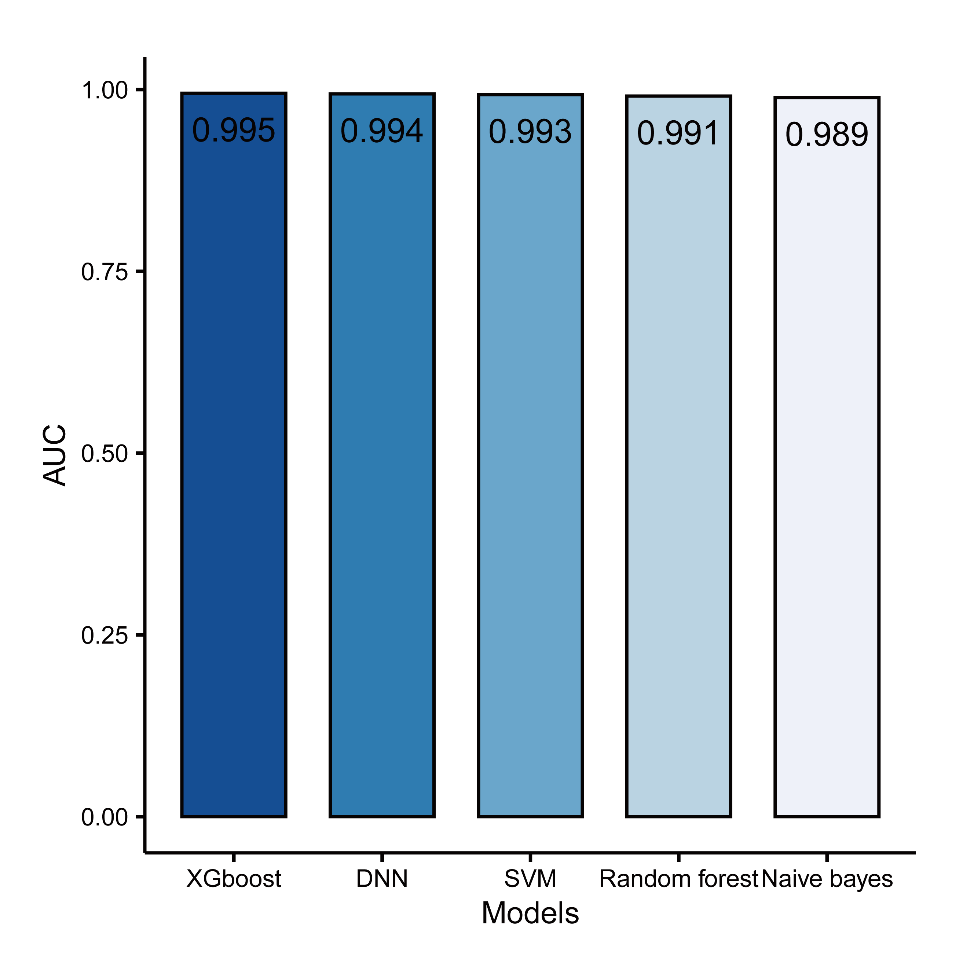


**Fig. S8 Performance evaluation of the models for predicting leaf development stage of maize based on AUC.**


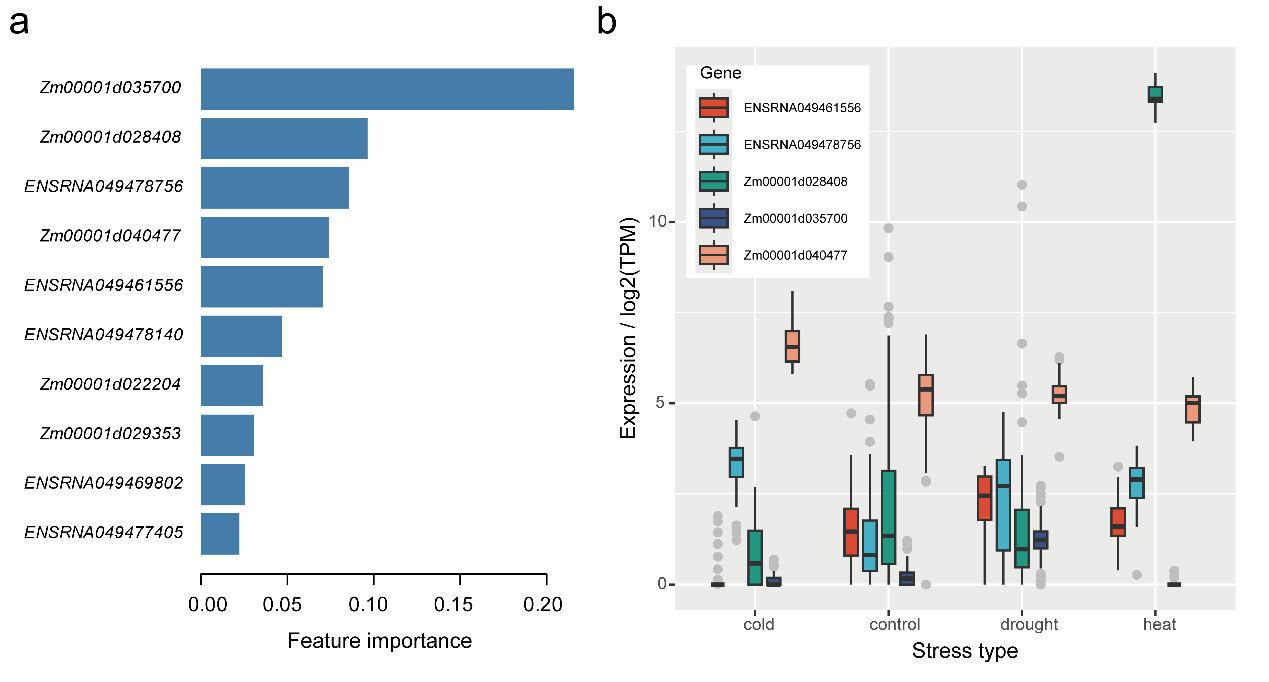
 **Fig. S9 Feature importance in prediction of stress types in maize using the XGboost model.** **(a)** Feature importance of the XGboost model. **(b)** Gene expression patterns of the top five features form the XGboost model.


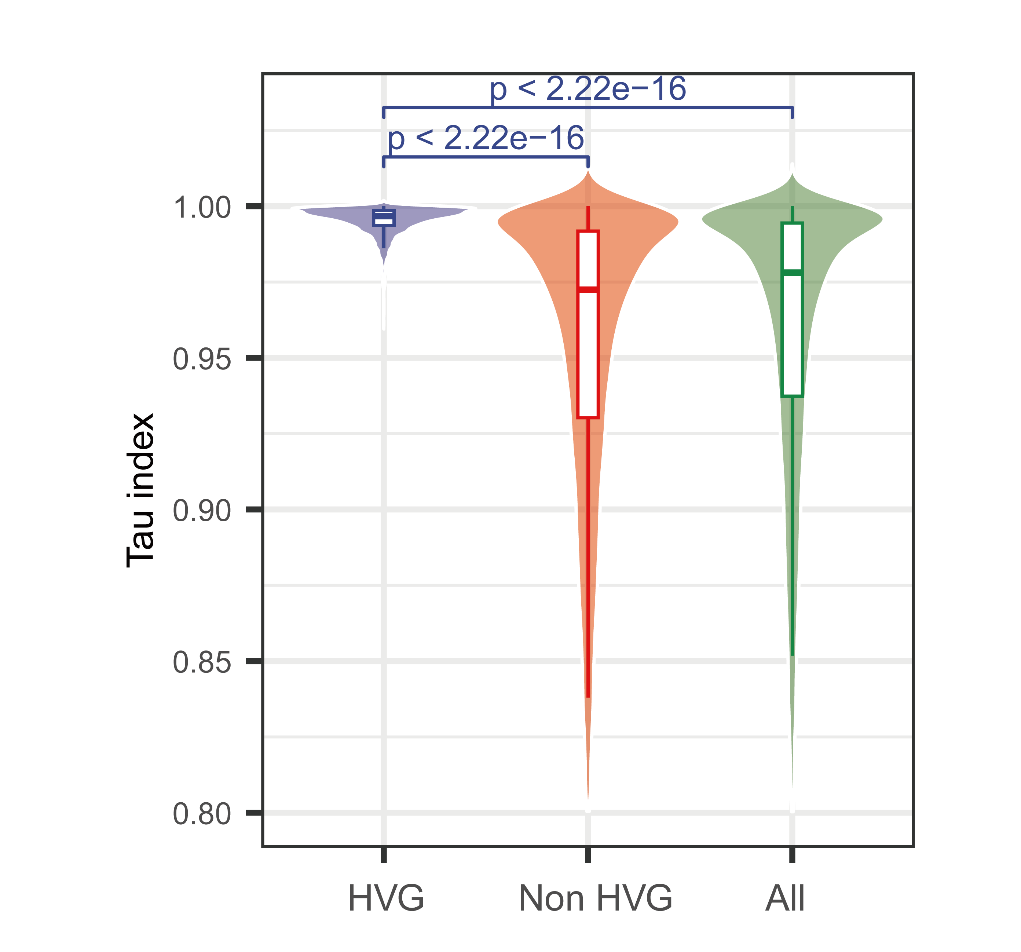


**Fig. S10 Gene expression specificity of rice HVGs.** HVG, highly variable gene. 'Non-HVG' represents all rice genes that do not belong to the HVGs. 'All' represents all gene models in rice. The statistical significance of differences between two groups was calculated with the Wilcoxon rank-sum test, and the *P* values are indicated in the figure


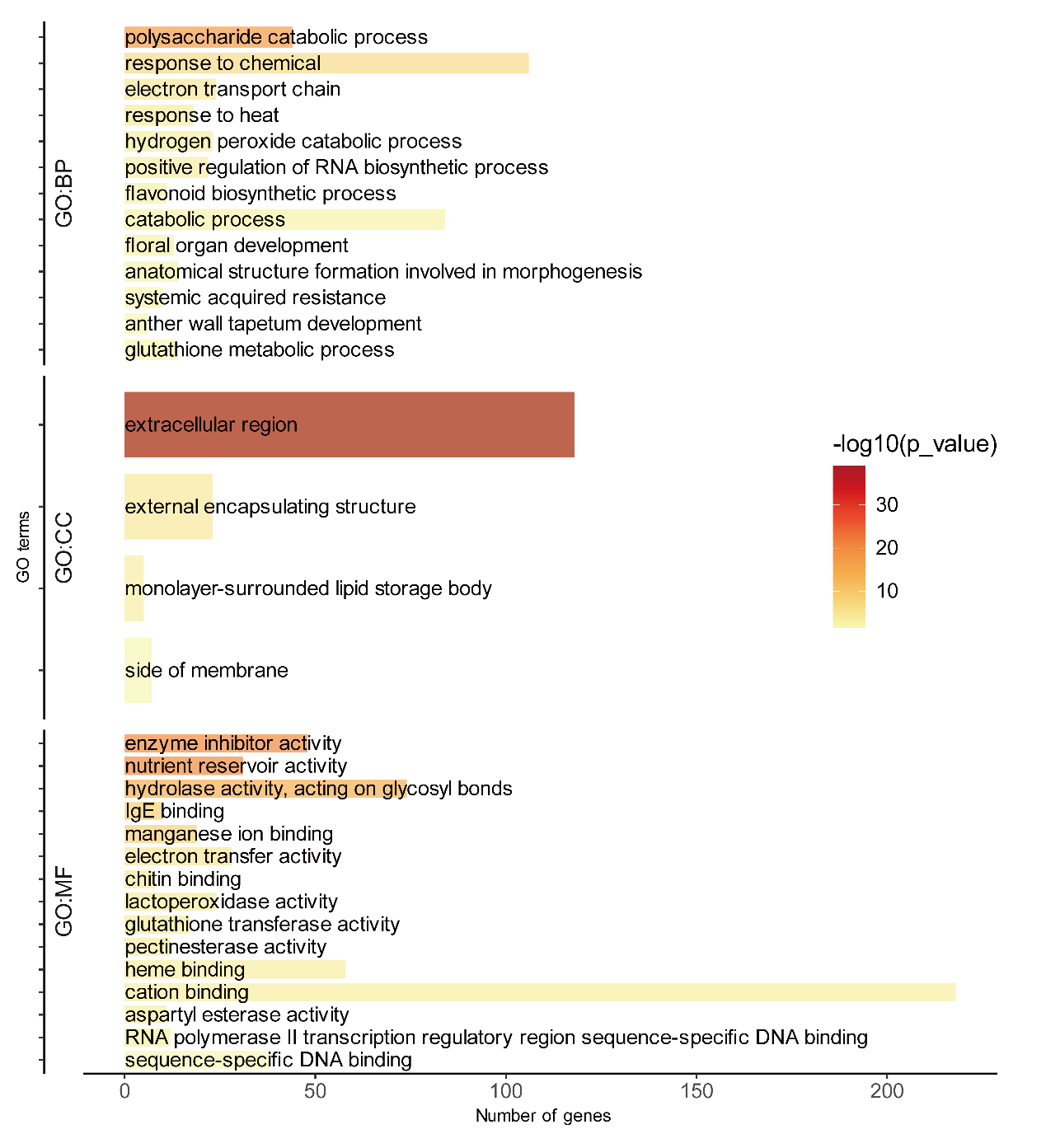


**Fig. S11 Gene function enrichment analysis of rice HVGs.** HVG, highly variable gene.
