# Supplementary material for: Phenotype prediction in plants is improved by integrating large-scale transcriptomic datasets
Source: NAR Genom Bioinform. 2024 Dec 27;6(4):lqae184. doi: 10.1093/nargab/lqae184 (PMC11672113; doi:10.1093/nargab/lqae184)
Supplement: lqae184_Supplemental_Files [file lqae184_supplemental_files.zip › Supplementary legend.docx]

## Supplementary Figures

**Fig. S1 Gene function enrichment analysis of maize HVGs.** HVG, highly variable gene.

**Fig. S2 Pie chart showing the number of maize RNA-Seq samples from different tissues.**

**Fig. S3 Workflow of phenotype prediction based on gene expression of HVGs.**

**Fig. S4 Evaluation of the model performance for tissue type prediction based on AUC values.**

**Fig. S5 Gene expression specificity of the top five features in predicting maize tissue types.**

**Fig. S6 Model performance using shuffled gene expression of maize HVGs.** HVG, highly variable gene.

**Fig. S7 Model performance using maize HVGs selected from the training dataset.** HVG, highly variable gene.

**Fig. S8 Performance evaluation of the models for predicting leaf development stage of maize based on AUC.**

**Fig. S9 Feature importance in prediction of stress types in maize using the XGboost model.**

**Fig. S10 Gene expression specificity of rice HVGs.** HVG, highly variable gene.

**Fig. S11 Function enrichment analysis of rice HVGs.** HVG, highly variable gene.

## Supplementary Data

**Supplementary Data 1. Summary of maize RNA-Seq samples used in this study.**

**Supplementary Data 2. Summary of rice RNA-Seq samples used in this study.**

**Supplementary Data 3. List of HVGs used to train machine learning models on maize.** HVG, highly variable gene.

**Supplementary Data 4. List of HVGs identified using the default parameters.**

**Supplementary Data 5. List of HVGs used to train machine learning models on rice.** HVG, highly variable gene.
